# A rare case of small cell neuroendocrine carcinoma of gallbladder origin

**DOI:** 10.1093/jscr/rjae386

**Published:** 2024-06-03

**Authors:** Uğur Can Dülger, Şahin Erdem, Emine Sena Cünük, Fatih Altıntoprak

**Affiliations:** Department of General Surgery, Faculty of Medicine, Sakarya University, Sakarya 51400, Sakarya, Turkey; Department of Pathology, Faculty of Medicine, Sakarya University, Sakarya 51400, Sakarya, Turkey; Department of General Surgery, Faculty of Medicine, Sakarya University, Sakarya 51400, Sakarya, Turkey; Department of General Surgery, Faculty of Medicine, Sakarya University, Sakarya 51400, Sakarya, Turkey

**Keywords:** neuroendocrine tumors, gallbladder, small cell neuroendocrine tumor

## Abstract

Neuroendocrine carcinomas (NECs) of the gallbladder are very rare and aggressive tumors with poor prognosis. Most of them are poorly differentiated and belong to the small cell type. We report a case of a 59-year-old woman who presented with abdominal pain and distension. Contrast-enhanced computed tomography revealed a large heterogeneous mass in the liver, adjacent to the gallbladder, and omental nodules. CA 19-9 level was elevated and ascitic fluid cytology was suspicious for malignancy. Percutaneous biopsy of the liver mass confirmed the diagnosis of small cell NEC of the gallbladder. The patient was considered inoperable and planned for chemotherapy, but she died 20 days after admission. This case illustrates the diagnostic challenges and the dismal outcome of small cell NEC of the gallbladder. Early detection and multimodal treatment are essential for improving the survival of these patients.

## Introduction

Neuroendocrine tumors are a rare group of tumors that originate from neuroendocrine cells located anywhere in the body and may have variable biological behaviors. They produce various hormones in the form of neuromodulators, neurotransmitters, and neuropeptides and may cause different clinical pictures or compression symptoms with mass effect. They are most commonly seen in the gastrointestinal and respiratory systems. The gallbladder is a very rare localization for neuroendocrine tumors and its prevalence has been reported to be only 0.21% of all gastrointestinal carcinoids [1, 2]. Small cell neuroendocrine tumors of the gallbladder are highly aggressive tumors with low survival rates and the literature is limited to a small number of cases. In this article, we present the clinical approach of a patient who was diagnosed with small cell neuroendocrine tumor of gallbladder origin and whose treatment process resulted in mortality [3].

## Case report

**Figure 1:**
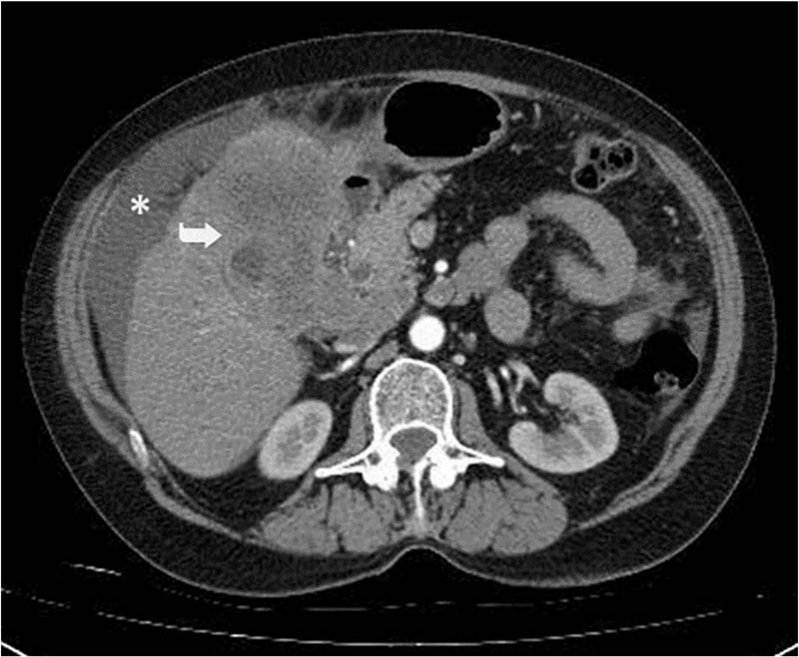
Irregular liver contours and perihepatic free fluid (*). Dense heterogeneous mass at the level of liver segment 4B-5, ~10×10 cm in area and extending to segment 6 (Arrow). Borders of the gallbladder could not be seen.

**Figure 2:**
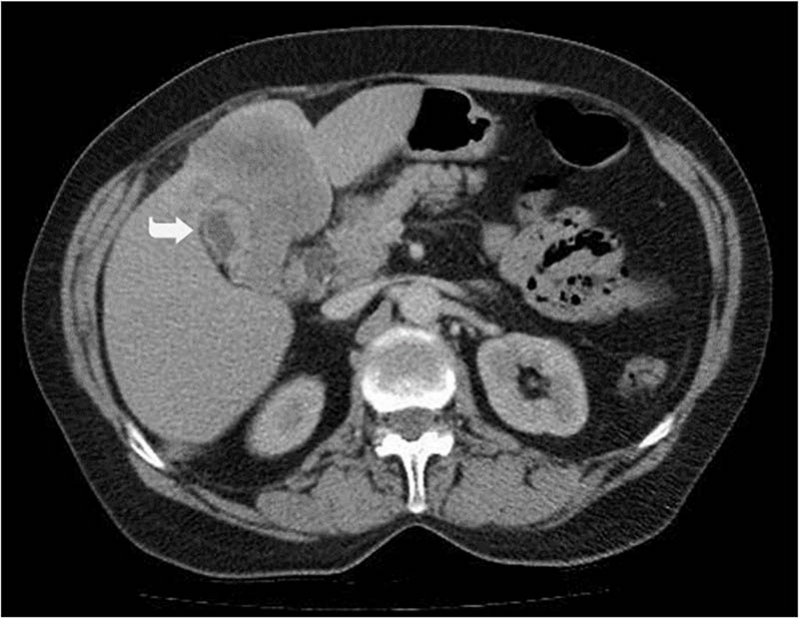
Hypodense lesions in the left lobe of the liver, 10×8 cm in size, protruding into the inferior perihepatic space and 3.5 cm in diameter in the right lobe segment 5 with no significant contrast enhancement after intravenous contrast material injection (Arrow).

**Figure 3:**
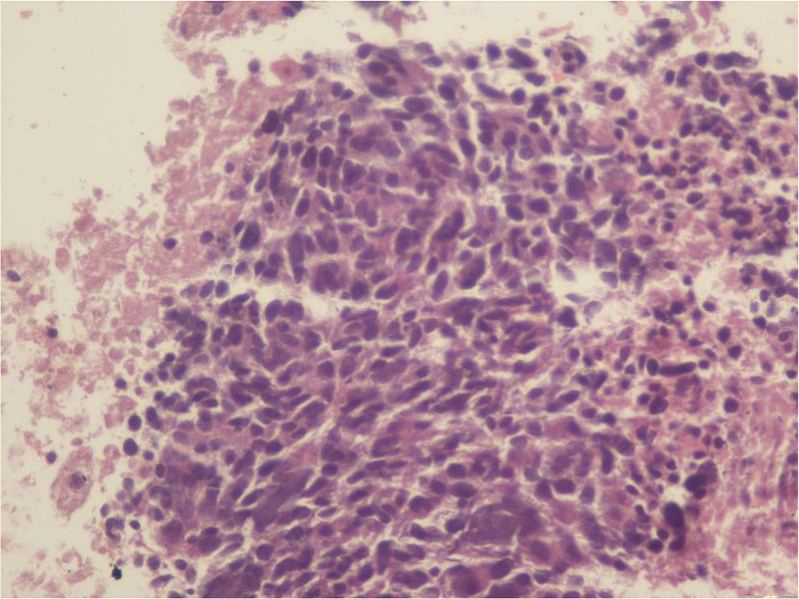
Monotonous tumor cells in a necrotic background (40×).

**Figure 4:**
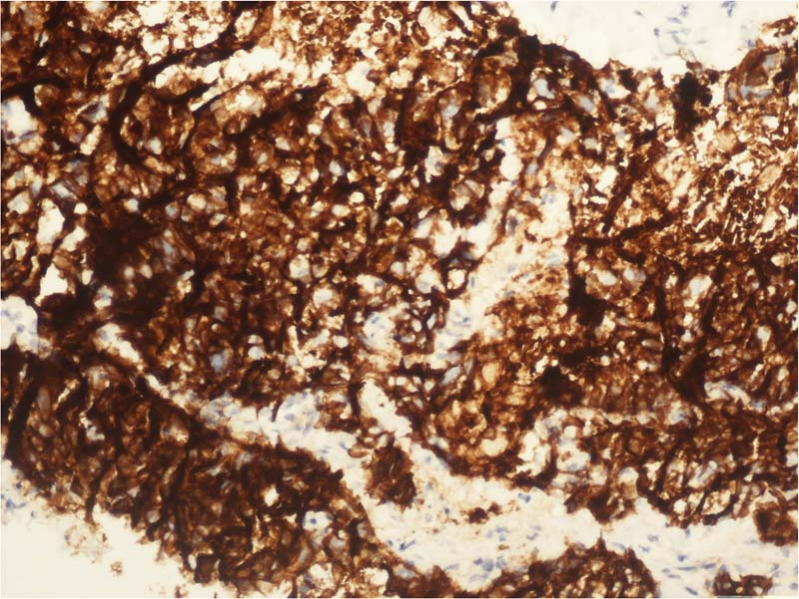
CD 56 positive immunohistochemical examination (20×).

**Figure 5:**
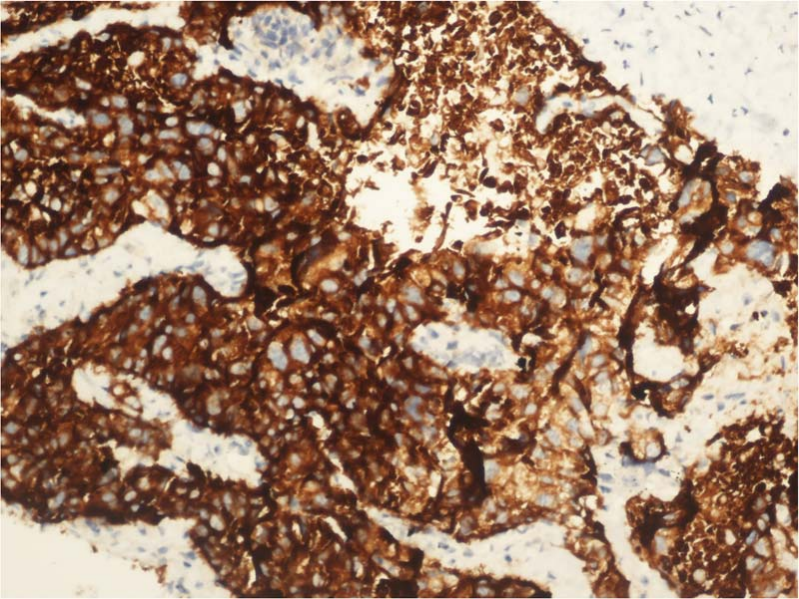
Synaptophysin positive immunohistochemical examination (20×).

**Figure 6:**
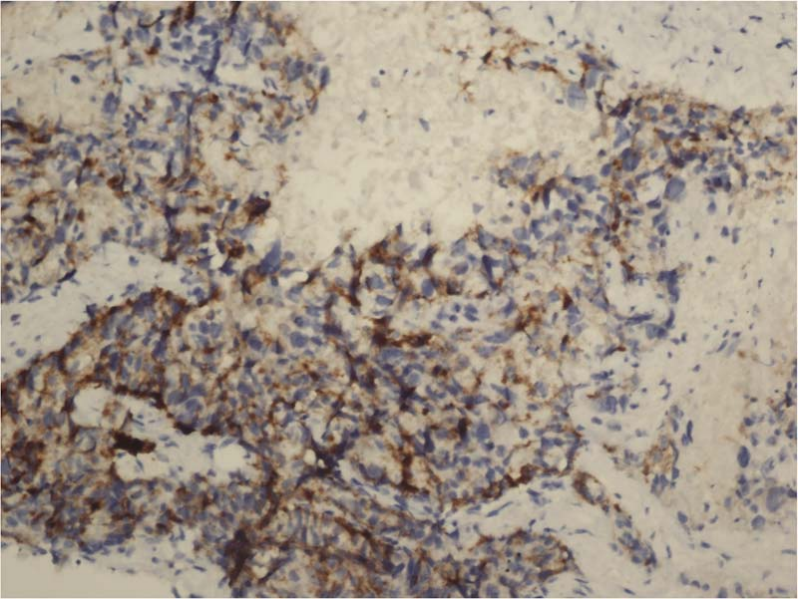
Chromogranin A positive immunohistochemical examination (20×).

**Figure 7:**
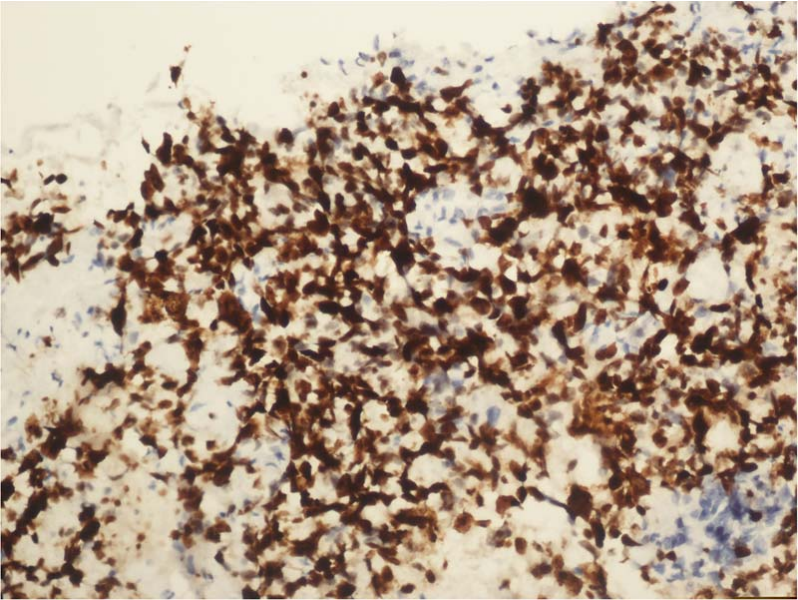
Immunohistochemical examination of Ki 67 index 90% (20×).

A 59-year-old woman presented with complaints of abdominal pain and abdominal distension for about a month. There was no known systemic disease and no history of previous abdominal surgery. It was learned that the patient had been admitted to the emergency department in another center one month ago with the current complaints and was discharged after medical treatment. Physical examination at the time of admission revealed abdominal distension and diffuse minimal tenderness with no signs of peritoneal irritation. Laboratory tests revealed that hemogram and biochemical parameters were within normal limits, but one of the tumor markers, the patient’s CA 19-9 level was significantly elevated at 855 U/ml (normal range: 0–37 U/ml). Contrast-enhanced CT scan showed a 10×10 cm heterogeneous mass in liver segments 4B-5, extending to segment 6, and free fluid adjacent to the liver and irregular liver contours. Borders of the gallbladder could not be seen. Soft tissues forming nodular mass formation were detected on the omental surfaces (Fig. 1). Computed tomography examination performed at the center where the patient was admitted as an emergency one month ago revealed hypodense lesions measuring 10×8 cm in size in the left lobe of the liver, protruding into the inferior perihepatic area, and 3.5 cm in diameter in segment 5 of the right lobe with no significant contrast enhancement after intravenous injection of contrast material. Perihepatic ascites was not detected and gallbladder size, wall thickness and lumen were normal (Fig. 2). In the light of the radiologic findings, a pre-diagnosis of progressive gallbladder malignancy was considered. The patient was hospitalized in the General Surgery ward for further investigation and treatment. Because of the presence of ascites causing abdominal distension, a percutaneous ascites drainage catheter was placed and 5000 cc ascites drainage was performed. Cytologic examination of the ascitic fluid revealed cell groups with unclear three-dimensional cytonuclear detail among lymphocytes and mesothelial cells with reactive atypia and malignancy was considered suspicious. With the current radiologic and cytologic findings, inoperable metastatic gallbladder carcinoma was considered. Medical treatment was decided by the multidisciplinary oncology council and 18G needle tru-cut biopsy was performed from the 10×10 cm mass adjacent to the gallbladder for definitive tissue diagnosis and adjuvant treatment planning. Histopathologic examination revealed a diagnosis of neuroendocrine carcinoma (NEC) (Fig. 3). Immunohistochemical examination revealed strong staining of tumor cells with CD56 (Fig. 4), synaptophysin (Fig. 5), chromogranin A (Fig. 6), and Ki67 index was >90% (Fig. 7). Weak staining was observed with CK19, CK7, and CDx2, but no staining was observed with CEA and CK20. Morphologic findings were compatible with small cell type. Etoposide and cisplatin chemotherapy was planned but the patient’s general condition deteriorated progressively. The patient couldn’t start chemotherapy and unfortunately resulted in mortality on the 20th day of hospitalization.

## Discussion

Small cell NEC is a rare and aggressive form of cancer that arises from neuroendocrine cells capable of producing hormones. It is characterized by rapid growth and a high potential for metastasis [1].

Small cell NEC most commonly occurs in the lungs, known as small cell lung cancer, but can also present in other organs such as the cervix, gastrointestinal tract, and pancreas. The incidence of small cell NEC varies depending on the primary site but is generally considered a rare entity [4].

The exact pathogenesis of small cell NEC is not fully understood; however, it is believed to originate from totipotent stem cells capable of neuroendocrine differentiation. These tumors are known for their high-grade malignancy and poor prognosis [5].

The diagnosis of small cell NEC typically involves a combination of clinical evaluation, imaging studies, and histopathological examination. Immunohistochemical staining is crucial for confirming neuroendocrine differentiation, with common markers including synaptophysin, chromogranin A, and CD56. A high Ki-67 index is indicative of the aggressive nature of small cell NEC [2].

Treatment strategies for small cell NEC include a combination of chemotherapy and radiotherapy. Surgical intervention may be considered in early-stage disease or for symptom management in advanced cases [6].

In this article, we present the clinical history of a 59-year-old woman with no systemic disease who went from being symptomatic to being diagnosed with gallbladder neuroendocrine tumor (NET) within a 2-month period to mortality before medical treatment could be initiated.

In a study evaluating the long-term survival of 18 cases of gallbladder neuroendocrine tumors, the median survival was 11 months [4]. In the literature, cases with healthy and recurrence-free follow-up at 2, 3, 8, and 18 months have been reported, as well as cases with excitus at 3 months after diagnosis [1–3, 6]. The symptoms for a month prior to the hospitalization and the 20 days admission of our patient, which was shorter than the other cases, supports the fact that gallbladder NET has a very poor prognosis as emphasized in previous studies [1, 3, 5]. The importance of early diagnosis and, if appropriate, effective surgical intervention in improving the prognosis of gallbladder NETs has been reported in previous studies [6]. In this context, the mortality of our case may be due to the difficulties in the diagnostic process. These difficulties have been emphasized in previous studies, such as the difficulty of diagnosing gallbladder NET with radiological imaging studies alone [7], the nonspecific clinical presentation symptoms, and the necessity of histopathological and immunocytochemical staining for definitive diagnosis [2]. The fact that the neuroendocrine tumor found in our patient was a small cell tumor may have led to a highly aggressive course with a poor prognosis, as seen in previous studies [1].
